# Emergence of SARS-CoV-2 Delta Variant, Benin, May–July 2021

**DOI:** 10.3201/eid2801.211909

**Published:** 2022-01

**Authors:** Anges Yadouleton, Anna-Lena Sander, Praise Adewumi, Edmilson F. de Oliveira Filho, Carine Tchibozo, Gildas Hounkanrin, Keke K. René, Dossou Ange, Rodrigue K. Kohoun, Ramalia Chabi Nari, Sourakatou Salifou, Raoul Saizonou, Clement G. Kakai, Sonia V. Bedié, Fattah Al Onifade, Michael Nagel, Melchior A. Joël Aïssi, Petas Akogbeto, Christian Drosten, Ben Wulf, Andres Moreira-Soto, Mamoudou Harouna Djingarey, Benjamin Hounkpatin, Jan Felix Drexler

**Affiliations:** Laboratoire des Fièvres Hémorragiques Virales du Benin, Cotonou, Benin (A. Yadouleton, P. Adewumi, C. Tchibozo, G. Hounkanrin, R.C. Nari);; Université Nationale des Sciences, Technologies, Ingénierie et Mathématiques, Abomey, Benin (A. Yadouleton, P. Adewumi, C. Tchibozo, G. Hounkanrin);; Charité–Universitätsmedizin Berlin, Berlin, Germany (A.-L. Sander, E.F. de Oliveira Filho, C. Drosten, B. Wulf, A. Moreira-Soto, J.F. Drexler);; Ministry of Health, Cotonou, Benin (K.K. René, D. Ange, R.K. Kohoun, S. Salifou, P. Akogbeto, B. Hounkpatin);; Organisation mondiale de la Santé Cotonou, Contonou (R. Saizonou, C.G. Kakai, S.V. Bedié, F. Al Onifade, M.A. Joël Aïssi, M.H. Djingarey);; Deutsche Gesellschaft für Internationale Zusammenarbeit, Bonn, Germany (M. Nagel);; German Centre for Infection Research, Berlin, Germany (C. Drosten, J.F. Drexler);; World Health Organization Regional Office for Africa, Brazzaville, Congo (M.H. Djingarey)

**Keywords:** COVID-19, respiratory infections, severe acute respiratory syndrome coronavirus 2, SARS-CoV-2, SARS, coronavirus disease, zoonoses, viruses, coronavirus, Delta variant, variant of concern, West Africa, Benin

## Abstract

Severe acute respiratory syndrome coronavirus 2 Delta variant epidemiology in Africa is unknown. We found Delta variant was introduced in Benin during April–May 2021 and became predominant within 2 months, after which a steep increase in reported coronavirus disease incidence occurred. Benin might require increased nonpharmaceutical interventions and vaccination coverage.

Numerous genetic variants of severe acute respiratory syndrome coronavirus 2 (SARS-CoV-2) have emerged globally since the start of the coronavirus disease (COVID-19) pandemic (https://cov-lineages.org). By September 2021, the World Health Organization defined 4 lineages as variants of concern (VOCs): B.1.1.7 (Alpha), B.1.351 (Beta), P.1 (Gamma), and B.1.617.2 (Delta) (*1*). The Delta VOC was detected in India in October 2020 (*1*). By September 2021, >33 sublineages (AY.1–AY.33) of the Delta VOC were reported globally (https://cov-lineages.org). Increased transmissibility of the Delta VOC compared with other lineages has been attributed to potential immune escape and intense replication (*2*; B. Li et al., unpub. data, https://www.medrxiv.org/content/10.1101/2021.07.07.21260122v2), which is consistent with the global spread of the Delta VOC (*1*), and rapid outcompetition of other lineages, such as Alpha and Kappa, in India (*3*). By August 2021, the Africa Centres for Disease Control and Prevention had reported Delta VOC infections from 30 countries (*4*). Nevertheless, epidemiologic information on the emergence and dissemination of the Delta VOC in Africa is missing. We conducted genomic surveillance to monitor emergence and spread of SARS-CoV-2 variants in Benin in West Africa.

## The Study

**Figure 1:**
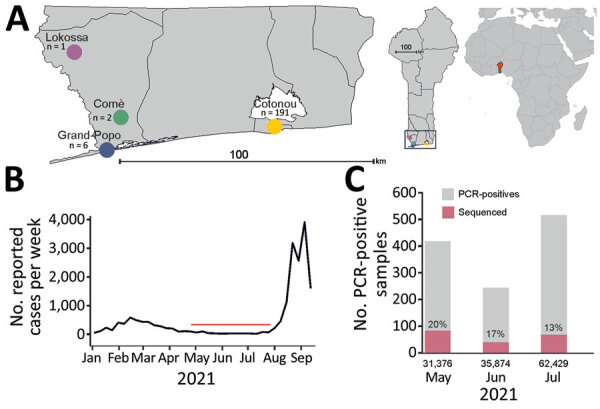
Molecular surveillance of severe acute respiratory syndrome coronavirus 2 (SARS-CoV-2) in Benin, May–July 2021. A) Sampling sites from which 200 SARS-CoV-2–positive respiratory samples were collected. Left map shows enlarged region of the 4 sampling sites in southern Benin; center map shows location sampling sites in the southern tip of the Benin; right map shows location of Benin (red) in Africa. Benin maps were obtained from The Humanitarian Data Exchange (https://data.humdata.org) and were plotted with the ggplot2 package in R (R Foundation for Statistical Computing, https://www.r-project.org); Africa map was generated by using the rworldmap package in R. B) Reported number of cases from Benin during 2021 based on data from the World Health Organization (https://covid19.who.int/region/afro/country/bj; accessed 2021 Sep 21). Red horizontal line indicates the study period. C) Monthly number of SARS-CoV-2–positive samples from the reference laboratory in Benin. Red denotes the number of samples that were sequenced in this study. Numbers below baseline indicate the total number of samples collected each month.

We recently described the circulation of 10 diverse SARS-CoV-2 lineages with mutations associated with VOCs in Benin (*5*), but we did not detect any Delta variants by the end of that study in late March 2021. Here, we report continuous genomic surveillance at the Benin reference laboratory on samples obtained from 4 sites in southern Benin during late April–mid-July 2021 (Figure 1, panel A). During the study period, routine testing at the reference laboratory and associated satellite laboratories in Benin averaged at 1,370 samples per day (Figure 1, panel B), a 900% increase from a comparable timeframe in 2020 (*6*) (Figure 1, panel B). The decentralization of diagnostic testing and simplification of extraction protocol contributed to increased testing (*7*).

**Figure 2:**
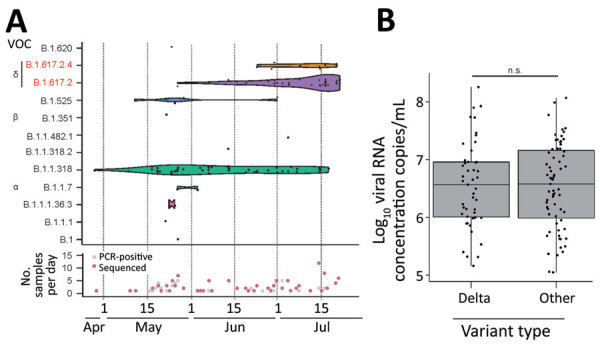
Virologic data on SARS-CoV-2 variants from respiratory samples, Benin, 2021. A) Top Gaussian kernel smoothed violins representing the density of observed occurrences per SARS-CoV-2 lineage at a given time point during the sampling period from the end of April until mid-July 2021. Black dots represent lineage occurrences of 114 generated genomes. Height of the violin plot corresponds to density of lineage in time. Bottom of graph shows collection date of the 200 SARS-CoV-2–positive samples collected for this study. Red indicates the subset for which near-full genomes were generated. Both plots were generated using the ggplot2 package in R (R Foundation for Statistical Computing, https://www.r-project.org). B) Log_10_ SARS-CoV-2 RNA concentrations of Delta variant strains versus all other lineages. Points represent each individual Log_10_ concentration. Box plots indicate interquartile range; whiskers represent the maximum and minimum values; horizontal line indicates the median. Plot was generated using the ggplot2 package in R. NS, not statistically significant by Student *t*-test; SARS-CoV-2, severe acute respiratory syndrome coronavirus 2; VOC, variant of concern.

During the study period, the laboratory identified 1,181 SARS-CoV-2–positive samples, specifically 419 in May, 245 in June, and 517 in July (Figure 1, panel C). For genomic surveillance, the laboratory kept 17.0% (200/1,181) of SARS-CoV-2–positive respiratory samples: 20% of all positive samples collected in May, 17% in June, and 13% in July (Figure 1, panel C). From those 200 samples, we selected 166 samples with a cycle threshold <35 by Sarbeco E-gene assay (TIB Molbiol, https://www.tib-molbiol.de) for genomic sequencing by using a previously described next-generation sequencing workflow (*5*). We attained near-complete viral genomes for 67.8% (114/166) of SARS-CoV-2–positive samples, 9.7% of all SARS-CoV-2–positive samples in Benin. The other 52 samples failed to yield sufficient genomic data for further analysis. Using Pangolin COVID-19 Lineage Assigner Version 3.0.2 (https://pangolin.cog-uk.io), we designated the 114 newly characterized SARS-CoV-2 genomes (Appendix Table 1) to 12 distinct lineages (Figure 2, panel A).

SARS-CoV-2 epidemiology in Benin changed drastically within just 2 months. The only commonality between our previous study (*5*) and this study was the continuous detection of the B.1.1.318 lineage and detection of the Alpha VOC. Unlike our previous study, we detected Beta and Delta variants in this study. Of note, we only sporadically detected Alpha and Beta VOCs, plus 6 other lineages, in this study (Figure 2, panel A), highlighting a lack of intense transmission of these variants during the study period. We first detected the Delta variant in a sample collected on May 27, 2021, in the capital of Benin, Cotonou (Appendix Table 2). To ensure that we did not miss any Delta strains during our previous study, we sequenced another 90 genomes (Appendix Table 1) collected during January–March 2021 but did not detect Delta VOCs in those samples.

Detection of the Delta variant increased rapidly, from 3.4% of positive samples in May to 37.9% in June and 63.6% in July (Table). We detected 2 sublineages of Delta variant, B.1.617.2 and AY.4, in our dataset. We first detected the Delta sublineage AY.4 in a sample collected in Cotonou on June 24, 2021, after which it co-circulated with the B.1.617.2 sublineage. Of note, we did not detect AY.4 outside of the capital city, Cotonou (Appendix Table 2). However, we cannot exclude a sampling bias because of low sample numbers from other cities, which could affect the apparent spatial distribution of the AY.4 sublineage. We did not detect Delta sublineage AY.1 signature mutations (V70F, W258L, and K417N) (*8*), nor did we detect the E484K mutation, which is associated with immune escape in the Delta sublineage AY.2 (*9*), in any of the Delta variant genomes from Benin (Appendix Figure 1). In addition, Delta VOC mutations did not occur at identical frequencies in SARS-CoV-2 strains circulating in Benin, (Appendix Figure 1), suggesting that the Delta variant expanded in Benin irrespective of mutations that are hypothesized to enhance partial immune escape.

**Table:** Detection rates of major SARS-CoV-2 lineages over time, Benin, May–mid-July 2021*

Pangolin lineages	% Lineages		
	May	June	July
B.1.1.7 Alpha	3.4	3.4	1.8
B.1.351 Beta	3.4	0	0
B.1.617.2 Delta	3.4	27.6	52.7
AY.4 Delta	0	10.3	10.9
B.1	3.4	0	0
B.1.1.1	3.4	0	0
B.1.1.318	58.6	55.2	29.1
B.1.525	13.8	0	1.8
B.1.620	3.4	0	0
AV.1	0	0	1.8
AZ.2	0	3.4	0
C.36.3	6.9	0	1.8

*Lineages according to Pangolin software (https://github.com/cov-lineages/pangolin). SARS-CoV-2, severe acute respiratory syndrome coronavirus 2.

In our study, samples containing Delta VOC strains did not show significantly higher viral RNA concentrations compared with other lineages (Figure 2, panel B). This observation contrasts preliminary studies showing higher concentrations of Delta VOCs in upper respiratory tract samples (B. Li et al., unpub. data, https://www.medrxiv.org/content/10.1101/2021.07.07.21260122v2). At the same time, those results countered a potential bias toward sequencing Delta VOC strains from putatively higher virus concentrations, exceeding the threshold we applied for genomic sequencing. We observed no differences in age or sex of patients infected with Delta versus non-Delta VOC strains, hinting at similar sociodemographic determinants of SARS-CoV-2 spread in Benin (Appendix Figure 2).

## Conclusions

Our data confirm that the SARS-CoV-2 Delta lineage was introduced into Benin during April–May 2021, ≈6 months after its detection in India and ≈1 month after its emergence in Europe (*10*). Reduced international connectivity in Benin likely delayed introduction of the Delta VOC (*7*). In addition, our data show that it took ≈2 months for Delta VOC strains to become predominant in Benin, which is roughly comparable to findings from India, where Delta became the predominant variant detected by genomic surveillance, with a frequency of 16% in March 2021 to 83% in April 2021 (https://nextstrain.org). Immediately after our study period, Benin reported a steep increase in COVID-19 cases to the World Health Organization (Figure 1, panel B), which could be associated with Delta VOC spread.

In India, Delta VOC takeover occurred at an average background SARS-CoV-2 seroprevalence of ≈50% by December 2020 (A. Velumani et al., unpub. data, https://www.medrxiv.org/content/10.1101/2021.03.19.21253429v1). Robust investigation of SARS-CoV-2 spread in Benin and adjacent countries could clarify whether Delta VOC spread occurred within largely naive or partially immune populations and elucidate potential immune escape by Delta VOC strains (*11*–*13*). Continentwide genomic surveillance should be pursued in Africa to assess the spread of SARS-CoV-2 VOCs, which would enable cross-national control measures and inform comparative studies (*11*; E. Wilkinson et al. unpub. data, https://www.medrxiv.org/content/10.1101/2021.05.12.21257080v1). However, direct comparisons between countries are challenging because of differences in vaccine coverage and socioeconomic factors, such as population density, connectivity, and wealth.

Our study is limited by a laboratory-based sampling that does not represent the total population of Benin. In addition, we cannot precisely define initial introduction of Delta VOCs into Benin because of the few samples collected during April 2021. Nonetheless, the steady increase of Delta VOC transmission and comparable speed and magnitude of Delta VOC takeover in other regions globally support the robustness of our data.

In conclusion, most COVID-19 vaccines protect against severe disease from Delta VOC infections (*14*). However, vaccination coverage in Benin is still only ≈1%, as in many other countries in Africa (https://www.bloomberg.com/graphics/covid-vaccine-tracker-global-distribution). Progress on vaccination campaigns will be crucial to limiting spread of the Delta VOC in countries in Africa.

Appendix Additional information on emergence of SARS-CoV-2 Delta variant, Benin, West Africa, May–July 2021.
