# Supplementary material for: Emergence of SARS-CoV-2 Delta Variant, Benin, May–July 2021
Source: Emerg Infect Dis. 2022 Jan;28(1):205–9. doi: 10.3201/eid2801.211909 (PMC8714210; doi:10.3201/eid2801.211909)
Supplement: Appendix — Additional information on emergence of SARS-CoV-2 Delta variant, Benin, West Africa, May–July 2021. [file 21-1909-Techapp-s1.pdf]

Article DOI: https://doi.org/10.3201/eid2801.211909

Emergence of SARS-CoV-2 Delta Variant, Benin, May–July 2021

Appendix

Appendix Table 1. GISAID accession numbers of SARS-CoV-2 genomes sequenced during a surveillance study of virus variants, Benin, 2021*

Sample collection dates	No. sequenced	GISAID accession nos.†
January–March	90	EPI_ISL_4567011, EPI_ISL_4567013, EPI_ISL_4567015, EPI_ISL_4567016, EPI_ISL_4567018, EPI_ISL_4567020, EPI_ISL_4567022, EPI_ISL_4567023, EPI_ISL_4567025, EPI_ISL_4567027, EPI_ISL_4567029, EPI_ISL_4567031, EPI_ISL_4567032, EPI_ISL_4567034, EPI_ISL_4567036, EPI_ISL_4567038, EPI_ISL_4567039, EPI_ISL_4567041, EPI_ISL_4567043, EPI_ISL_4567045, EPI_ISL_4567046, EPI_ISL_4567048, EPI_ISL_4567050, EPI_ISL_4567052, EPI_ISL_4567054, EPI_ISL_4567056, EPI_ISL_4567058, EPI_ISL_4567059, EPI_ISL_4567061, EPI_ISL_4567063, EPI_ISL_4567065, EPI_ISL_4567067, EPI_ISL_4567069, EPI_ISL_4567071, EPI_ISL_4567072, EPI_ISL_4567074, EPI_ISL_4567076, EPI_ISL_4567078, EPI_ISL_4567080, EPI_ISL_4567081, EPI_ISL_4567083, EPI_ISL_4567085, EPI_ISL_4567087, EPI_ISL_4567089, EPI_ISL_4567090, EPI_ISL_4567092, EPI_ISL_4567094, EPI_ISL_4567096, EPI_ISL_4567098, EPI_ISL_4567099, EPI_ISL_4567101, EPI_ISL_4567103, EPI_ISL_4567105, EPI_ISL_4567107, EPI_ISL_4567108, EPI_ISL_4567110, EPI_ISL_4567112, EPI_ISL_4567114, EPI_ISL_4567116, EPI_ISL_4567118, EPI_ISL_4567120, EPI_ISL_4567122, EPI_ISL_4567124, EPI_ISL_4567126, EPI_ISL_4567128, EPI_ISL_4567130, EPI_ISL_4567132, EPI_ISL_4567133, EPI_ISL_4567135, EPI_ISL_4567137, EPI_ISL_4567139, EPI_ISL_4567141, EPI_ISL_4567143, EPI_ISL_4567144, EPI_ISL_4567146, EPI_ISL_4567148, EPI_ISL_4567150, EPI_ISL_4567152, EPI_ISL_4567153, EPI_ISL_4567155, EPI_ISL_4567157, EPI_ISL_4567158, EPI_ISL_4567160, EPI_ISL_4567162, EPI_ISL_4567164, EPI_ISL_4567166, EPI_ISL_4567168, EPI_ISL_4567169
April–July	114	EPI_ISL_4566811, EPI_ISL_4566813, EPI_ISL_4566814, EPI_ISL_4566816, EPI_ISL_4566818, EPI_ISL_4566820, EPI_ISL_4566821, EPI_ISL_4566823, EPI_ISL_4566825, EPI_ISL_4566827, EPI_ISL_4566829, EPI_ISL_4566830, EPI_ISL_4566832, EPI_ISL_4566834, EPI_ISL_4566836, EPI_ISL_4566837, EPI_ISL_4566839, EPI_ISL_4566841, EPI_ISL_4566843, EPI_ISL_4566845, EPI_ISL_4566846, EPI_ISL_4566848, EPI_ISL_4566850, EPI_ISL_4566852, EPI_ISL_4566854, EPI_ISL_4566855, EPI_ISL_4566857, EPI_ISL_4566859, EPI_ISL_4566861, EPI_ISL_4566863, EPI_ISL_4566865, EPI_ISL_4566867, EPI_ISL_4566869, EPI_ISL_4566871, EPI_ISL_4566872, EPI_ISL_4566874, EPI_ISL_4566876, EPI_ISL_4566878, EPI_ISL_4566880, EPI_ISL_4566882, EPI_ISL_4566883, EPI_ISL_4566885, EPI_ISL_4566887, EPI_ISL_4566889, EPI_ISL_4566891, EPI_ISL_4566893, EPI_ISL_4566894, EPI_ISL_4566896, EPI_ISL_4566898, EPI_ISL_4566900, EPI_ISL_4566901, EPI_ISL_4566903, EPI_ISL_4566905, EPI_ISL_4566907, EPI_ISL_4566908, EPI_ISL_4566910, EPI_ISL_4566912, EPI_ISL_4566914, EPI_ISL_4566915, EPI_ISL_4566917, EPI_ISL_4566919, EPI_ISL_4566921, EPI_ISL_4566922, EPI_ISL_4566924, EPI_ISL_4566926, EPI_ISL_4566928, EPI_ISL_4566929, EPI_ISL_4566931, EPI_ISL_4566933, EPI_ISL_4566935, EPI_ISL_4566936, EPI_ISL_4566938, EPI_ISL_4566940, EPI_ISL_4566942, EPI_ISL_4566943, EPI_ISL_4566945, EPI_ISL_4566947, EPI_ISL_4566949, EPI_ISL_4566950, EPI_ISL_4566952, EPI_ISL_4566954, EPI_ISL_4566956, EPI_ISL_4566958, EPI_ISL_4566959, EPI_ISL_4566961, EPI_ISL_4566963, EPI_ISL_4566964, EPI_ISL_4566966, EPI_ISL_4566968, EPI_ISL_4566970, EPI_ISL_4566971, EPI_ISL_4566973, EPI_ISL_4566975, EPI_ISL_4566977, EPI_ISL_4566978, EPI_ISL_4566980, EPI_ISL_4566982, EPI_ISL_4566984, EPI_ISL_4566985, EPI_ISL_4566987, EPI_ISL_4566989, EPI_ISL_4566990, EPI_ISL_4566992, EPI_ISL_4566994, EPI_ISL_4566996, EPI_ISL_4566997, EPI_ISL_4566999, EPI_ISL_4567001, EPI_ISL_4567002, EPI_ISL_4567004, EPI_ISL_4567006, EPI_ISL_4567008, EPI_ISL_4567009, EPI_ISL_4572438

*SARS-CoV-2, severe acute respiratory syndrome coronavirus 2.
†GISAID, https://www.gisaid.org.

Appendix Table 2. Designated lineages, number of samples, and sample location and dates of 114 detected SARS-CoV-2 genomes, Benin, May–July 2021*

Lineage	WHO label	No. samples	Alias	All locations detected	Earliest sample	
					Date collected	Location
B.1.1.318	NA	50	NA	Cotonou, Come, Grand-Popo	28.04.2021	Grand-Popo
B.1.617.2	Delta	38	NA	Cotonou, Come, Lokossa	27.05.2021	Cotonou
AY.4	Delta sublineage	9	B.1.617.2.4	Cotonou	24.06.2021	Cotonou
B.1.525	NA	5	NA	Cotonou, Grand-Popo	12.05.2021	Grand-Popo
B.1.1.7	Alpha	3	NA	Cotonou	27.05.2021	Cotonou
C.36.3	NA	3	B.1.1.1.36.3	Cotonou	24.05.2021	Cotonou
AV.1	NA	1	NA	Cotonou	05.07.2021	Cotonou
AZ.2	NA	1	NA	Cotonou	14.06.2021	Cotonou
B.1	NA	1	NA	Cotonou	27.05.2021	Cotonou
B.1.1.1	NA	1	NA	Cotonou	23.05.2021	Cotonou
B.1.351	Beta	1	NA	Cotonou	23.05.2021	Cotonou
B.1.620	NA	1	NA	Cotonou	25.05.2021	Cotonou

*NA, not applicable; SARS-CoV-2, severe acute respiratory syndrome coronavirus 2; WHO, World Health Organization.

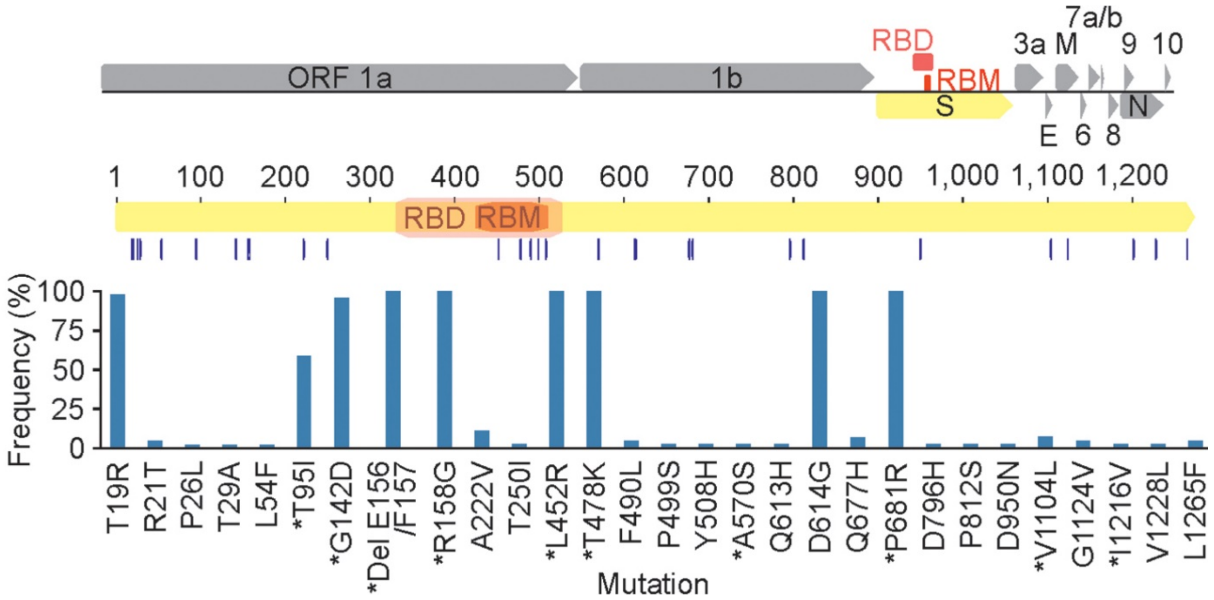

Appendix Figure 1. Nonsynonymous substitutions within the spike protein occurring within 47 SARS-CoV-2 Delta variant genomes sampled, Benin, May–July 2021. For clarity, a SARS-CoV-2 full genome illustration is included at the top. Asterisks (*) indicate mutations for which genomic information was not available in all 47 Delta variant genomes, potentially due to assay failures or relatively lower DNA concentrations. Blue bars represent frequency of a given mutation in 46 genomes (for T95I, L452R, T478K, and P681R mutations); in 45 genomes (for A570S, V1140L, and I1216V mutations); and in 23 genomes (for G142D, Del E156/F157, and R158G mutations).

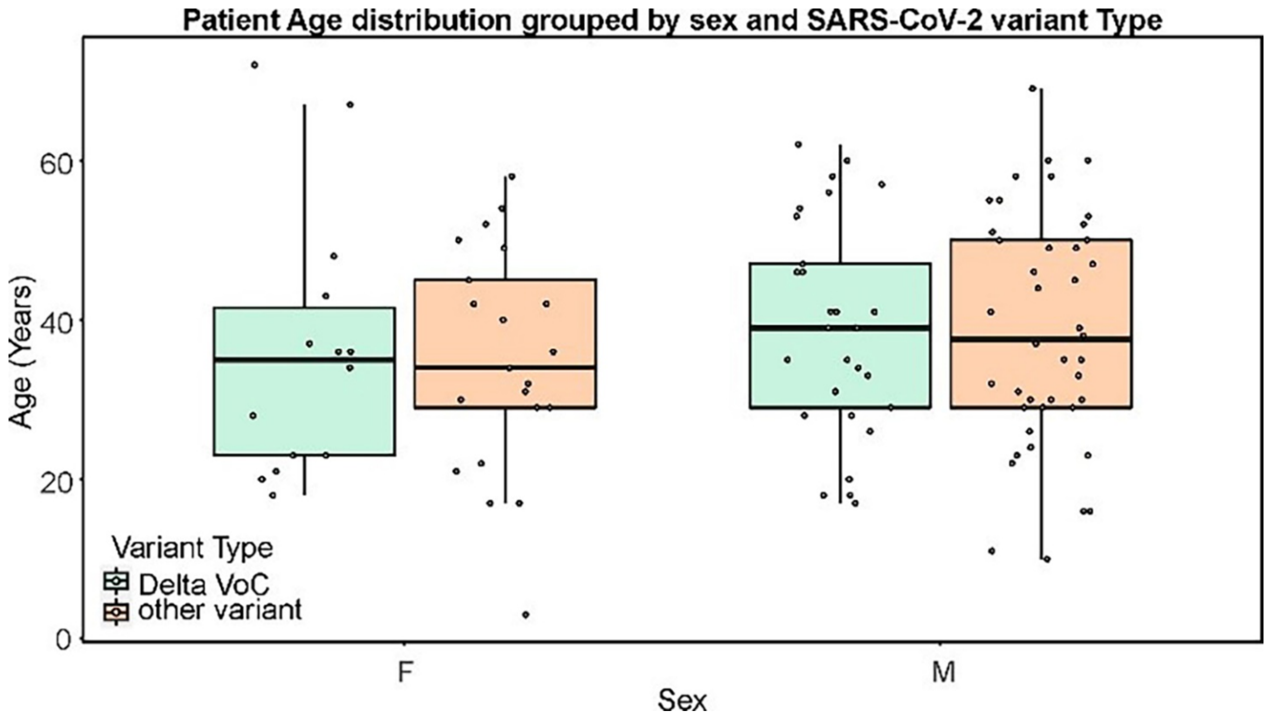

Appendix Figure 2. Patient age distribution grouped by sex and SARS-CoV-2 variant type, Benin, May–July 2021. Each point represents 1 patient. Box plots indicate interquartile range; whiskers represent the maximum and minimum values; horizontal line indicates the median. Plot was generated using the ggplot2 package in R (R Foundation for Statistical Computing, https://www.r-project.org).
